# 慢病毒介导的稳定沉默*nm23-H1*基因的肺癌细胞株的建立及生物学行为改变

**DOI:** 10.3779/j.issn.1009-3419.2012.03.02

**Published:** 2012-03-20

**Authors:** 猛 罗, 大兴 朱, 磊 弓, 小明 邱, 玲玲 祖, 丽亚 孙, 志浩 吴, 清华 周

**Affiliations:** 1 300052 天津，天津市肺癌转移与肿瘤微环境重点实验室，天津市肺癌研究所，天津医科大学总医院 Tianjin Key Laboratory of Lung Cancer Metastasis and Tumor Microenvironment, Tianjin Lung Cancer Institute, Tianjin Medical University General Hospital, Tianjin 300052, China; 2 550002 贵阳，贵州省人民医院胸外科 Department of Thoracic Surgery, the Hospital of Guizhou Province, Guiyang 550002, China

**Keywords:** 肺肿瘤, 慢病毒, RNA, 基因, Lung neoplasms, Lentivirus, RNA, Genes

## Abstract

**背景与目的:**

*nm23-H1*基因是重要的肿瘤转移抑制基因。前期研究发现利用化学合成的小干扰RNA（small interfering RNA, siRNA）抑制*nm23-H1*基因的表达可明显增强肺癌细胞的侵袭力。为了进一步研究*nm23-H1*基因沉默后的分子生物学机制，本研究利用慢病毒介导的短发夹RNA（short hairpin RNA, shRNA）建立*nm23-H1*基因稳定沉默的肺癌细胞株。

**方法:**

将表达特异性抑制*nm23-H1*基因shRNA的慢病毒转染人大细胞肺癌细胞株NL9980和肺腺癌细胞株A549，通过嘌呤霉素筛选出稳定转染细胞株。逆转录PCR、定量PCR及Western blot法检测*nm23-H1*基因表达，并通过shRNA抵抗的*nm23-H1*基因重组质粒转染拯救实验验证，侵袭小室实验检测侵袭力改变。

**结果:**

逆转录PCR、定量PCR和Western blot法检测稳定转染细胞株NL9980-99和A549-99中*nm23-H1*基因在mRNA和蛋白水平表达均明显降低；shRNA抵抗的*nm23-H1*基因重组质粒转染拯救实验重现*nm23-H1*的正常表达；侵袭小室实验显示NL9980-99和A549-99细胞侵袭力明显增强。

**结论:**

成功建立*nm23-H1*基因稳定沉默的人大细胞肺癌细胞株NL9980-99和人肺腺癌细胞株A549-99，*nm23-H1*基因沉默后使NL9980-99和A549-99细胞的侵袭力明显增强。

## Abstract (en)

**Background and objective:**

The *nm23-H1* gene is an important tumor metastatic suppressor gene. Our previous study showed that the downregulation of *nm23-H1* gene expression using small interfering RNA (siRNA) in NL9980 lung cancer cells greatly enhanced their invasiveness. To further explore the molecular mechanisms after *nm23-H1* gene knockdown, we established transgene NL9980 and A549 lung cancer cell lines with stable *nm23-H1* gene silencing through the lentivirus-mediated short hairpin RNA (shRNA) method.

**Methods:**

The human large cell lung cancer NL9980 and human lung adenocarcinoma A549 cells were transfected with shRNA lentiviral particles specific for the *nm23-H1* gene, and were then selected through puromycin. Puromycin-resistant clones were generated and screened using reverse transcription polymerase chain reaction (RT-PCR), quantitative real-time polymerase chain reaction (qPCR), and Western blot analysis. shRNA rescue experiments were performed to restore the *nm23-H1* gene expression in the shRNA-expressing cells. Invasiveness was determined through a Boyden chamber assay.

**Results:**

The puromycin-resistant clones (NL9980-99 and A549-99) showed very low levels of *nm23-H1* mRNA and protein expression under RT-PCR, qPCR, and Western blot analysis. Meanwhile, the shRNA rescue experiment restored the *nm23-H1* expression in the NL9980-99 and A549-99 cells detected by Western blot. Downregulation of *nm23-H1* gene expression enhanced the invasiveness of the NL9980-99 and A549-99 cells compared with the controls.

**Conclusion:**

The lung cancer cell lines NL9980-99 and A549-99 with stable *nm23-H1* gene silencing were successfully established and their invasiveness was greatly increased after *nm23-H1* gene knockdown.

肿瘤侵袭转移是一个多基因调控、多阶段、多步骤发生的复杂过程，也是导致肿瘤患者治疗失败和死亡的主要原因。*nm23-H1*基因是第一个被发现的重要的肿瘤转移抑制基因，它的结构和功能的异常与癌细胞的侵袭和转移能力密切相关^[1-5]^。在前期工作中将化学合成的siRNA瞬时转染低转移的人大细胞肺癌细胞株NL9980以抑制*nm23-H1*基因的表达，研究发现NL9980细胞的侵袭力明显增强^[6]^。但化学合成的siRNA介导的基因沉默只能维持较短的时间（5 d-7 d），为了更深入地研究*nm23-H1*基因沉默后对肺癌细胞侵袭转移机制的影响，本研究利用慢病毒介导的shRNA技术，转染人大细胞肺癌细胞株NL9980和肺腺癌细胞株A549，建立*nm23-H1*基因稳定沉默的细胞株，并观察其体外侵袭力的改变，为进一步研究*nm23-H1*的功能、生化作用机制奠定基础。

## 1 材料与方法

### 1.1 材料

人大细胞肺癌细胞株NL9980由本实验室建立。pcDNA3.1（+）表达载体为本实验保存。A549肺腺癌细胞株购自美国ATCC。RPMI-1640、小牛血清购自美国Gibco公司。非靶标shRNA对照慢病毒颗粒（non-targeting shRNA, SHC002V）和特异性抑制*nm23-H1*基因的shRNA慢病毒颗粒（nm23-H1-shRNA；Clone ID：NM_000269.x-99s1c1、NM_000269.x-182s1c1和NM_000269.x-183s1c1）、嘌呤霉素和聚凝胺购自美国Sigma公司。克隆环购自美国Corning公司。M-MLV逆转录酶、dNTP mix、RNase抑制剂、随机引物、DNA Marker购自TAKARA公司。总RNA提取试剂Trizol Reagent购自美国Invitrogen公司。引物、Gold view核酸染料购自北京赛百盛公司。SYBR GREEN Master Mix、7500实时定量PCR系统购自美国ABI公司。nm23-H1鼠单抗（货号：SC-56928）购自美国Santa Cruz公司，β-actin鼠单抗（货号：A5441）购自美国Sigma公司。Boyden小室购自美国MiliPore公司。

### 1.2 方法

#### 1.2.1 shRNA构建、慢病毒包装

慢病毒载体为pLKO.1-puro，由U6启动子引导shRNA的合成，并具有嘌呤霉素抗性筛选标记。将特异性靶向nm23-H1（NM_000269.2）mRNA不同序列（NM_000269.x-99s1c1：GCGTACCTTCATTGCGATCAA、NM_000269.x-182s1c1：TCCGCCTTGTTGGTCTGAAAT和NM_000269.x-183s1c1：CCGCCTTGTTGGTCTGAAATT）的21 bp反向互补发夹序列克隆入pLKO.1-puro载体，并包装生产慢病毒，病毒滴度为10^6^ TU，购自Sigma公司。

#### 1.2.2 细胞培养、慢病毒转染和克隆细胞株筛选

转染前1天将2×10^5^个NL9980和A549细胞铺板（6孔板），第2天细胞融合度为50%-60%，每孔加入含终浓度为8μg/mL的聚凝胺和慢病毒50 µL转染。转染24 h后加入嘌呤霉素1 µg/mL（选择浓度由杀菌曲线确定）。每隔2 d-3 d观察细胞情况，更换选择培养基。2周左右开始有克隆生长。克隆环方法挑取单克隆细胞入24孔板，90%-100%融合度后转入培养瓶扩增，鉴定，保种。

#### 1.2.3 逆转录和定量PCR检测*nm23-H1*基因表达

根据总RNA提取试剂盒说明书提取RNA，并进行逆转录PCR。逆转录nm23-H1引物：Forward：5′CAAGTGCTGCGAACCACG3′；Reverse：5′GACCAACAAGGCGGAATC3′，扩增长度为420 bp。参照基因*GAPDH*引物序列：Forward：5′ATGGGGAAGGTGAAGGTCG3′；Reverse：5′GGGGTCATTGATGGCAACAATA3′，扩增长度为108 bp。PCR扩增条件为：94 ℃、3 min，94 ℃、30 s，55 ℃、30 s，72 ℃、2 min，30个循环；72 ℃、5 min。取PCR产物10 μL进行2%琼脂糖凝胶电泳检测。nm23-H1定量PCR引物：Forward：5′AAAGGATTCCGCCTTGTTGGT3′；Reverse：5′GCCCTGAGTGCATGTATTTCAC3′，扩增长度为124 bp。定量PCR条件为：50 ℃、2 min、1个循环；95 ℃、10 min、1个循环；95 ℃、15 s，60 ℃、1 min，40个循环。做熔解曲线检测。2^-ΔΔCT^法计算基因表达差异^[7]^。

#### 1.2.4 Western blot印迹检测nm23-H1表达的变化

细胞裂解后提取总蛋白，BCA方法测细胞蛋白浓度后进行12% SDS-PAGE电泳。100 V转膜1 h，抗nm23-H1和β-actin一抗4 ℃孵育过夜，洗膜后室温二抗1 h。ECL显影。

#### 1.2.5 shRNA抵抗*nm23-H1*基因重组质粒转染拯救实验

根据shRNA（NM_000269.x-99s1c1: GCGTACCTTCATTGCGATCAA）序列设计*nm23-H1*基因shRNA抵抗序列，下划线标记为突变碱基，该突变为沉默突变，不改变nm23-H1氨基酸编码序列。突变引物：Forward：5′CGGATCCATGGCCAACTGTGAGCGAACATTTATCGCCATCAAACCA3′；Reverse: 5′GCTCTAGATCATTCATAGATCCAGTTCTGA3′。表达载体为pcDNA3.1（+）。PCR突变构建shRNA抵抗*nm23-H1*基因重组质粒。脂质体法将shRNA抵抗*nm23-H1*基因重组质粒3 µg转染稳定沉默*nm23-H1*基因表达的肺癌细胞株NL9980-99和A549-99，48 h后收获细胞做Western blot检测。

#### 1.2.6 侵袭小室实验检测侵袭力改变

每个上室加300 µL无血清的RPMI-1640进行ECM胶水化1.5 h，下室加500 µL含10%胎牛血清的RPMI-1640；然后将实验组和对照组细胞接种于Transwell 6孔板的上室中，每孔接种细胞5×10^5^，每组设3个平行孔。在细胞培养箱孵育48 h，对下室细胞消化后细胞计数，以穿过膜的细胞数目来表示肿瘤细胞的侵袭能力。

### 1.3 统计学处理

采用SPSS 13.0软件分析系统处理结果，计量资料采用Mean±SD表示，应用*t*检验分析数据。*P* < 0.05为差异有统计学意义。

## 2 结果

### 2.1 *nm23-H1*基因稳定沉默细胞系mRNA和蛋白表达水平的检测

人大细胞肺癌细胞株NL9980细胞用慢病毒介导的nm23-H1 shRNA转染、筛选后，Western blot检测3个shRNA（Clone ID: NM_000269.x-99s1c1, NM_000269.x-182s1c1和NM_000269.x-183s1c1）均获得很好的*nm23-H1*基因沉默效果，以shRNA（NM_000269.x-99s1c1）最为明显，而非靶向干扰序列则对nm23-H1的表达没有抑制作用。故在A549肺癌细胞筛选实验中只选用shRNA（NM_000269.x-99s1c1）病毒颗粒作转染，并将*nm23-H1*基因稳定沉默的人大细胞肺癌细胞株NL9980和肺腺癌细胞株A549命名为NL9980-99和A549-99，将非靶标（non-target）对照细胞株命名为NL9980-non和A549-non。RT-PCR结果显示与对照细胞株相比NL9980-99细胞*nm23-H1*基因含量明显降低（图 1A），A549-99细胞株实时定量PCR结果显示*nm23-H1*基因mRNA表达水平明显降低（*P*=0.008, 501）（图 1B），Western blot结果均证实nm23-H1蛋白表达明显抑制（图 1C，图 1D）。

**1:**
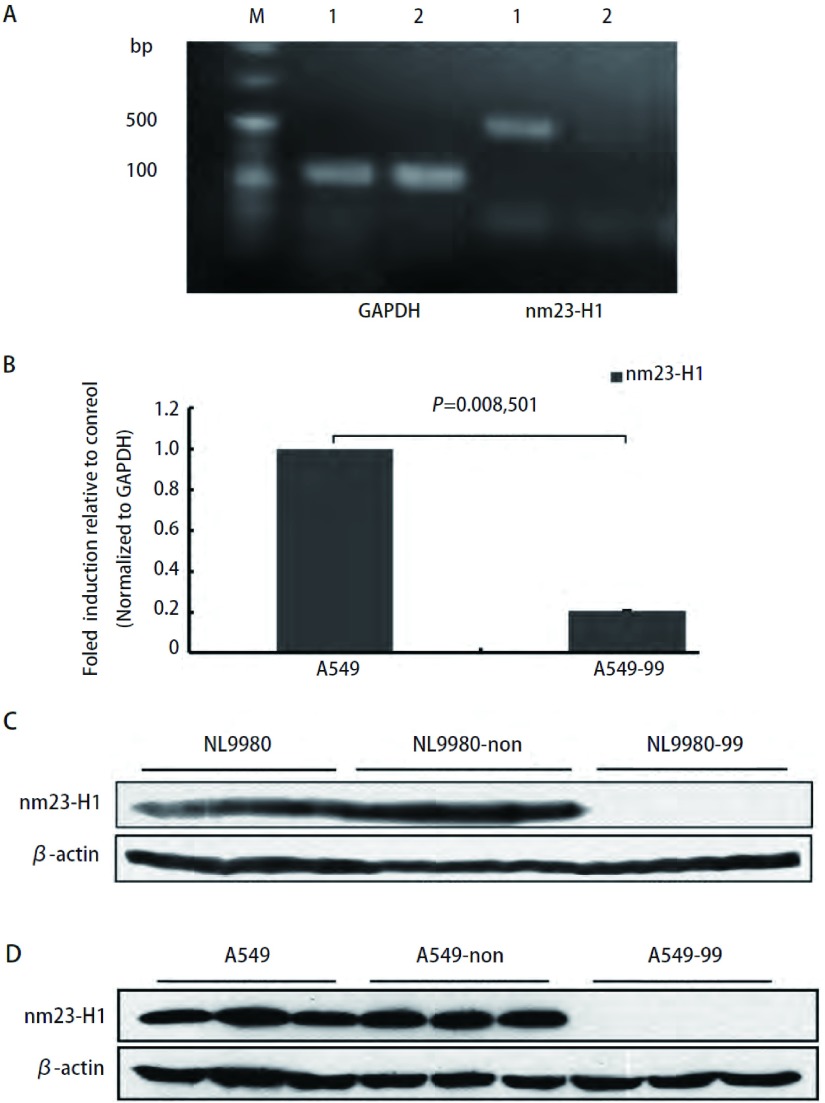
RT-PCR、定量PCR及Western blot检测结果。A：RT-PCR检测nm23-H1 mRNA在NL9980-99细胞中的表达，相对内参为GAPDH，与对照相比nm23-H1含量明显降低；1：NL9980；2：NL9980-99；B：qRT-PCR检测nm23-H1 mRNA在A549-99细胞中的表达，相对内参为GAPDH（*P* < 0.01）；C：Western blot检测nm23-H1蛋白在NL9980-99细胞中的表达，与对照相比表达量明显降低；D：Western blot检测nm23-H1蛋白在A549-99细胞中的表达，与对照相比表达量明显降低。 Results of RT-PCR, qRT-PCR and Western blot. A: The results of nm23-H1 mRNA RT-PCR, nm23-H1 expression greatly reduced compared with GAPDH; M: DNA marker; 1: NL9980; 2: NL9980-99; B: The results of nm23-H1 mRNA qRT-PCR, analysis of expression normalized with GAPDH (*P* < 0.01); C: The results of nm23-H1 expression in NL9980-99 cells by Western blot, nm23-H1 expression greatly reduced compared with *β*-actin; D: The results of nm23-H1 expression in A549-99 cells by Western blot, nm23-H1 expression greatly reduced compared with *β*-actin.

### 2.2 shRNA抵抗*nm23-H1*基因重组质粒转染拯救实验

将shRNA抵抗*nm23-H1*基因重组质粒转染稳定沉默*nm23-H1*基因表达的肺癌细胞株NL9980-99和A549-99，48 h后收获细胞做Western blot检测，显示恢复nm23-H1的正常表达（图 2A，图 2B）。

**2:**
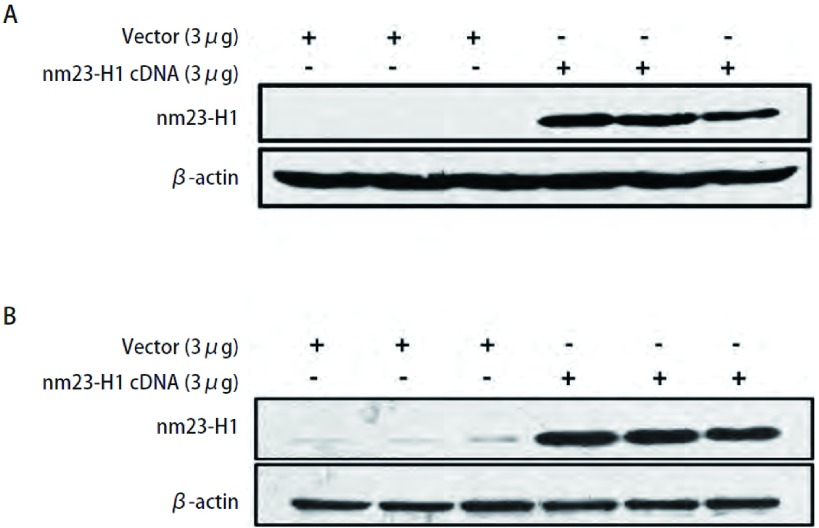
NL9980-99和A549-99细胞shRNA抵抗*nm23-H1*基因重组质粒转染拯救实验。A：NL9980-99细胞*nm23-H1*基因shRNA抵抗拯救实验，与对照组相比*nm23-H1*基因重组质粒转染组重现nm23-H1的正常表达；B：A549-99细胞*nm23-H1*基因shRNA抵抗拯救实验，与对照组相比*nm23-H1*基因重组质粒转染组重现nm23-H1的正常表达。 shRNA rescue experiments in NL9980-99 and A549-99 cells. NL9980-99 and A549-99 cells were transfected with vector containing shRNA-resistant nm23-H1 cDNA to rescue the expression of nm23-H1. A: shRNA rescue experiments in NL9980-99 cells analyzed by Western blot; B: shRNA rescue experiments in A549-99 cells analyzed by Western blot.

### 2.3 *nm23-H1*基因表达抑制后侵袭力改变

Boyden小室实验检测发现*nm23-H1*基因稳定沉默后，NL9980-99细胞和A549-99细胞体外侵袭能力明显增强，与对照组NL9980、NL9980-non和A549、A549-non细胞比较有统计学差异（*P* < 0.01），提示*nm23-H1*基因稳定沉默促进肺癌细胞的侵袭能力（图 3A，图 3B）。

**3:**
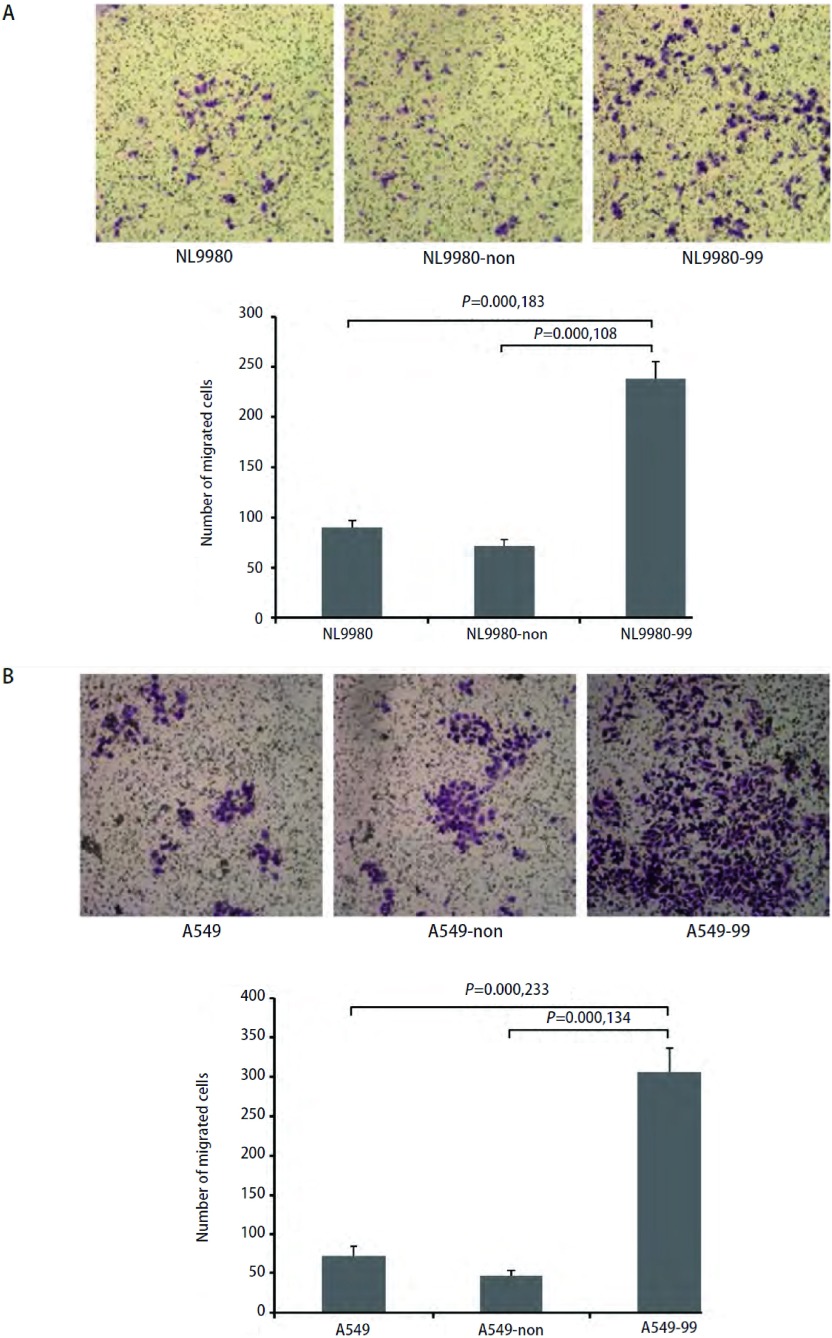
Boyden小室检测NL9980-99和A549-99细胞侵袭力的改变。A：NL9980-99细胞与对照相比，侵袭力明显增强（*P* < 0.01）；B：A549-99细胞与对照相比，侵袭力明显增强（*P* < 0.01）。 Boyden chamber assay of NL9980-99 and A549-99 cells. A: Boyden chamber assay of NL9980-99 cells, compared with the control (*P* < 0.01); B: Boyden chamber assay of A549-99 cells, compared with the control (*P* < 0.01).

## 3 讨论

*nm23*基因是第一个被发现的肿瘤转移抑制基因^[1]^。迄今为止，人类*nm23*基因已报道了8个亚型，即nm23-H1至nm23-H8，其中*nm23-H1*基因与肿瘤侵袭转移的关系最密切，已得到人们的普遍重视^[8]^。nm23-H1具有二磷酸核苷激酶（nucleotide diphosphate kinase, NDPK）活性^[9]^、组氨酸蛋白激酶活性^[10]^和3’-5’核酸外切酶活性^[11]^。近期研究^[12]^显示nm23-H1参与了紫外线诱导的DNA损伤修复过程，并可能与紫外线诱发的黑色素瘤的形成有关。Conery等^[13]^报道nm23-H1表达的缺失可以导致细胞染色体不稳定，从而参与肿瘤的形成过程。尽管20多年来国内外做了大量的研究，*nm23-H1*基因抑制肿瘤侵袭转移的分子机制还未完全阐明。

我们的前期研究^[14-16]^显示*nm23-H1*基因的低表达和杂合性缺失与人肺癌的高转移性有密切关系。将野生型*nm23-H1*基因转染人高转移大细胞肺癌细胞株L9981可以逆转L9981的侵袭、转移表型^[17]^。将化学合成的siRNA瞬时转染低转移的人大细胞肺癌细胞株NL9980抑制*nm23-H1*基因的表达，NL9980细胞的侵袭力明显增强；通过基因芯片检测发现基因表达谱发生明显变化，表达上调的基因有707个，下调的有373个。其中上调基因主要有肿瘤转移相关基因、细胞增殖、细胞周期、生长发育（包括胚胎发育和神经系统发育）以及细胞运动迁徙相关基因；下调基因包括肿瘤抑制基因、细胞骨架相关基因等^[6]^。上述研究结果说明*nm23-H1*基因可能是肿瘤侵袭转移的上游调控基因，但*nm23-H1*基因调控的关键下游分子或靶点尚需进一步研究确定。

为了进一步研究这些功能改变及相关的分子机制，需要建立稳定沉默*nm23-H1*基因的肺癌细胞株。RNA干扰技术现已成为研究基因功能的重要工具^[18]^，但化学合成的siRNA介导的基因沉默只能维持较短的时间（5 d-7 d），而且通常转染的效率较低^[19]^。因此，通过载体介导的RNA干扰就成为了选择。近年来，由于慢病毒技术的发展及其自身的优点，如免疫原性低、感染范围广，可以高效整合到宿主细胞基因组稳定产生siRNA，使得慢病毒介导的RNA干扰成为稳定基因沉默的最常用选择，并可克服化学合成siRNA的缺点^[20]^。

本研究通过慢病毒介导的特异性靶向*nm23-H1*基因shRNA，转染人大细胞肺癌细胞株NL9980和肺腺癌细胞株A549，经过嘌呤霉素筛选获得了稳定沉默*nm23-H1*基因表达的肺癌细胞株NL9980-99和A549-99。mRNA和蛋白水平检测均证实nm23-H1的表达明显降低，并通过转染shRNA抵抗的nm23-H1表达载体，恢复nm23-H1的表达。该拯救实验证实我们所建立的NL9980-99和A549-99细胞中*nm23-H1*基因沉默是由于RNA干扰机制降解了*nm23-H1*基因mRNA所致，而不是脱靶效应（off-target effect）^[19]^。通过Boyden小室实验观察到*nm23-H1*基因沉默后，人大细胞肺癌细胞株NL9980和人肺腺癌细胞株A549细胞的侵袭力明显增强，差异具有统计学意义（*P* < 0.01）。不同肺癌细胞株*nm23-H1*基因沉默后具有相似的侵袭力改变，逆向证明了*nm23-H1*基因肿瘤转移抑制的功能。以上结果显示本课题组成功建立了*nm23-H1*基因稳定沉默的人大细胞肺癌细胞株NL9980-99和肺腺癌细胞株A549-99，为更深入研究nm23-H1的功能、生化作用机制奠定了基础。
